# A 176 amino acid polypeptide derived from the mumps virus HN ectodomain shows immunological and biological properties similar to the HN protein

**DOI:** 10.1186/1743-422X-7-195

**Published:** 2010-08-20

**Authors:** Emma Herrera, Patricia Barcenas, Rubicela Hernández, Alfonso Méndez, Guillermo Pérez-Ishiwara, Blanca Barrón

**Affiliations:** 1 Lab Virología, ENCB-IPN Carpio y Plan de Ayala S/N Casco de Santo Tomás, México D.F. 11340 México; 2 Depto. de Bioquímica, ENCB-IPN Carpio y Plan de Ayala S/N Casco de Santo Tomás, México D.F. 11340 México; 3 Biomedicina Molecular, ENMyH-IPN, Guillermo Massieu Helguera Núm. 239, Frac. La Escalera México, D.F. 07320 México

## Abstract

**Background:**

The hemagglutinin-neuraminidase (HN) protein is the major antigenic determinant of the Mumps virus (MuV) and plays an important role in the viral infectious cycle through its hemagglutination/hemadsorption (HA/HD) and neuraminidase (NA) activities. *Objective*: analyze the biological and immunological properties of a polypeptide derived from a highly conserved region of the HN ectodomain. *Methods*: a highly conserved region of the HN gene among several MuV genotypes was chosen to be cloned in a eukaryotic expression vector. The pcDNAHN176-construct was transfected into Vero cells and RNA expression was detected by RT-PCR, while the corresponding polypeptide was detected by immunofluorescence and immunochemistry techniques. The HD and NA activities were also measured. The immunogenic properties of the construct were evaluated using two systems: rabbit immunization to obtain sera for detection of the HN protein and neutralization of MuV infection, and hamster immunization to evaluate protection against MuV infection.

**Results:**

A 567 nucleotide region from the HN gene was amplified and cloned into the plasmid pcDNA3.1. Vero cells transfected with the construct expressed a polypeptide that was recognized by a MuV-hyperimmune serum. The construct-transfected cells showed HD and NA activities. Sera from immunized rabbits *in vitro *neutralized two different MuV genotypes and also detected both the HN protein and the HN176 polypeptide by western blot. Hamsters immunized with the pcDNAHN176-construct and challenged with MuV showed a mild viral infection in comparison to non-immunized animals, and Th1 and Th2 cytokines were detected in them.

**Conclusions:**

The pcDNAHN176-construct was capable of expressing a polypeptide in Vero cells that was identified by a hyperimmune serum anti Mumps virus, and these cells showed the HD and NA activities of the complete MuV HN protein. The construct also elicited a specific immune response against MuV infection in hamsters.

## Background

Mumps is generally a childhood illness characterized by parotid gland inflammation caused by the mumps virus (MuV). The disease is usually mild, and approximately one-third of MuV infections are asymptomatic. However, up to 10% of patients may develop aseptic meningitis and other less frequent, but more serious, complications, such as encephalitis, deafness, orchitis and pancreatitis, which can result in permanent disability. In fact, mumps encephalitis accounted for 36% of the total viral encephalitis cases before introduction of the MuV vaccine [1-7]. It has been accepted that MuV is a monotypic virus [8]. However, this assumption has been challenged due to the recent resurgence of mumps epidemics in many countries with ongoing vaccination programs [9-13], the presence of several mumps reinfection cases [14], along with the evidence of distinct lineages of MuV co-circulating globally [6,11,13,15-20]. Currently, thirteen MuV genotypes (A to M) have been defined on the basis of the nucleotide sequence of the MuV SH gene [6,10,21]. Furthermore, two important mumps outbreaks were recently reported, one in 2005 in the UK, and the other in 2006 in the USA. In both cases, the G MuV genotype was identified, even though both countries have been using the mumps Jeryl Lynn vaccine, which has been identified as an A genotype [5,6,22].

MuV is a member of the genus *Rubulavirus *of the *Paramyxoviridae *Family. Its genome is a single-stranded, negative sense, non-segmented RNA of 15,384 nucleotides. The genome encodes for three nucleocapsid-associated proteins: an RNA binding protein (N), a phosphoprotein (P) and a large polymerase protein (L), four membrane proteins, an unglycosylated inner membrane or matrix protein (M) and three glycosylated envelope proteins, the fusion protein (F), the hemagglutinin-neuraminidase (HN) protein and the small hydrophobic protein (SH) [23].

HN is the major antigenic protein known to elicit neutralizing antibodies [23]. It also plays an important role in the viral infectious cycle. It is the viral attachment protein for host cell receptors (sialylated glycoconjugates), enhances the fusogenic activity of the viral F protein to allow viral entry into the cell, and its sialidase activity hydrolyzes sialic acid residues to prevent virus self-aggregation, facilitating viral spread of the new virions [24].

The crucial role played by the HN protein in the host protective immune response against MuV infections makes this protein a good target to develop a vaccine that might be useful against most of the MuV genotypes. Therefore, the aim of this paper was to look for a highly conserved and immunogenic region of the HN protein among different mumps virus genotypes and express the corresponding polypeptide. By *in silico *analyses, a highly conserved region of the HN gene among different MuV genotypes was found and this paper describes construction of the DNA recombinant vector and biological characterization of the expressed polypeptide.

## Results

### Characterization of the pcDNAHN176-construct

**Figure 1:**
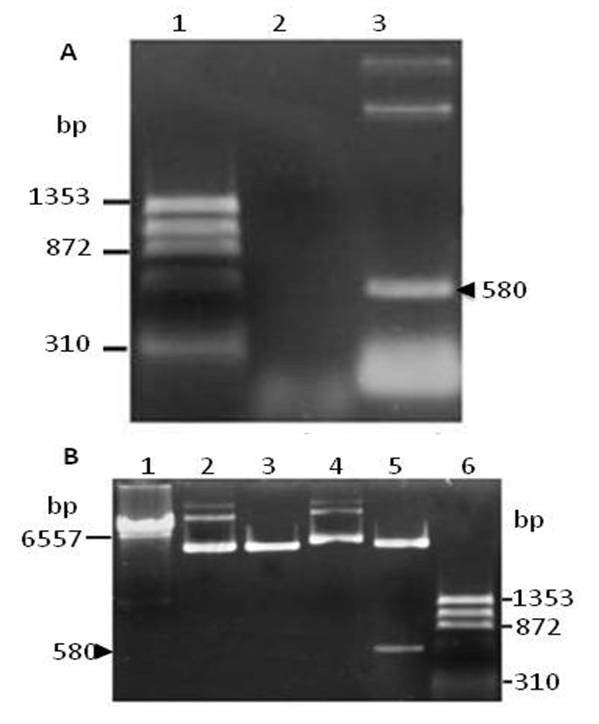
**Characterization of the pcDNAHN176-construct by PCR and enzymatic restriction**. **A) **PCR amplification of the insert using the HN primers. Lane 1) ϕX174 DNA-*HaeIII *marker; Lane 2) Negative control; Lane 3) pcDNA-HN176. The arrow indicates the amplicon of 580 bp. 1% agarose gel/100 V/1 hr/49 mA. **B) **Enzymatic restriction with *Bam HI *and *KpnI *to release the HN176 insert. Lane 1) λ Hind III marker; Lane 2) Unrestricted pcDNA3.1; Lane 3) Restricted pcDNA3.1; Lane 4) Unrestricted pcDNA-HN176; Lane 5) restricted pcDNA-HN176, Lane 6) ϕX174 DNA-*HaeIII *marker. The arrow indicates the insertion of 567 bp. 1% agarose gel/100 V/1 hr/45 mA

The PCR amplification of the pcDNAHN176-construct using the set of HN primers initially designed produced a 580-bp fragment, which corresponded to the expected size of the HN insert (Figure 1A, lane 3). Enzymatic restriction of the pcDNAHN176-construct released a 567-bp fragment, which was the size of the HN gene fragment previously cloned (Figure 1B, lane 5). Sequencing of the HN gene fragment indicated that the insert could encode for a 176 amino acid polypeptide (aa 255-431) that shared a 97.3% similarity with the Urabe HN protein (data not shown).

### Expression of the HN176 fragment in Vero transformed cells

**Figure 2:**
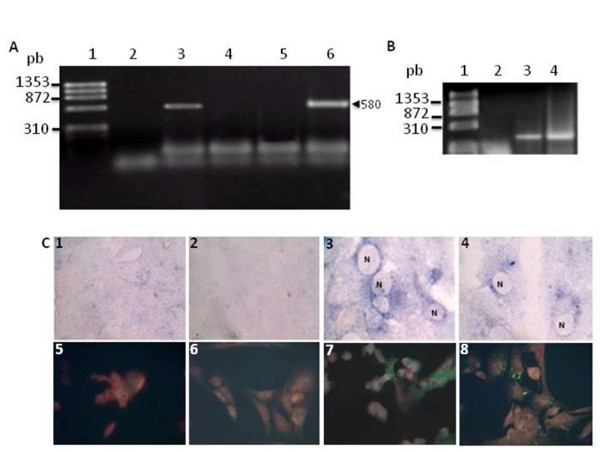
**Expression of the pcDNAHN176-construct**. **A) **Detection of HN176 mRNA in transfected Vero cells by RT-PCR amplification. Lane 1) ϕX174 DNA-*HaeIII *marker; Lane 2) RT/PCR negative control; Lane 3) Positive control (MuV-infected cells); Lane 4) Vero-untransfected cells, 5) Vero cells transfected with the plasmid pcDNA3.1, Lane 6) Vero cells transfected with the pcDNA-HN176-construct. The arrow indicates 580 bp amplicon. 1% agarose gel/100 V/1 hr/49 mA. **B**) RT-PCR Controls. Lane 1) ϕX174 DNA-*HaeIII *marker; Lane 2) PCR amplification of the RNA samples obtained from pcDNAHN176-transfected Vero cells without a previous RT reaction; Lane 3 and 4) RT/PCR amplification of β-actin gene using RNA samples obtained from HeLa cells and pcDNAHN176-transfected Vero cells, respectively. **C**) Immunodetection of the HN176 polypeptide by immunochemistry (1^st ^row) and immunofluorescence (2^nd ^row). Frames 1 & 5 mock infected cells; 2 & 6 pcDNA3.1-transfected cells; 3 & 7 MuV-infected cells; 4 & 8 pcDNAHN176-transfected cells, the blue (immunochemistry) and the green (immunofluorescence) colors indicate a positive reaction, N indicates the nucleus. 40×

The RNA-HN176 expression was analyzed by RT-PCR using total RNA extracted from pcDNAHN176-transfected Vero cells. A 580-bp specific band (Figure 2A, lane 6) that corresponded to the expected size of HN insert was detected. No amplification was obtained from untransfected Vero cells or from cells transfected with the pcDNA3.1 vector (Figure 2A, lanes 4 and 5). To verify that the 580 bp amplicon obtained from pcDNAHN176-transfected cells was not due to an amplification of the DNA construct, total RNA was directly used as a template for PCR, and no amplification was observed (Figure 2B, lane 2).

The expression of HN polypeptide was evaluated by immunochemistry and immunofluorescence assays using a hyperimmune anti-MuV serum. Both assays showed that the pcDNAHN176-transfected cells reacted with the anti-MuV serum, even though their reactivity was lower compared to MuV-infected cells (Figure 2C).

### Biological activities of the HN176 polypeptide

**Figure 3:**
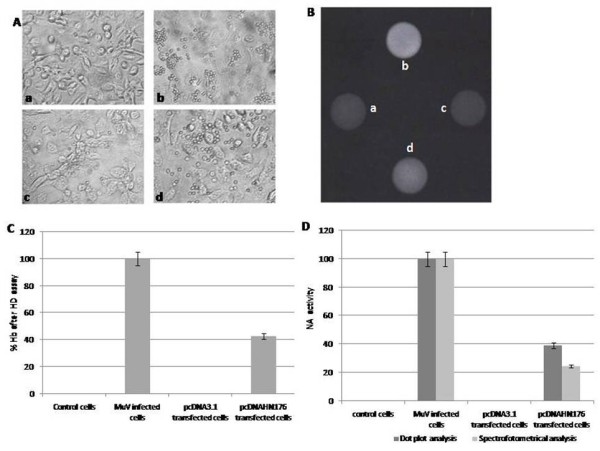
**Hemadsorption and neuraminidase activities in the pcDNAHN176 transfected cells**. **A)**. Hemadsorption (HD) reaction. a) Mock infected cells; b) MuV-infected cells; c) pcDNA-3.1-transfected cells; d) pcDNAHN176-transfected Vero cells. The red cell aggregates indicate a positive HD. 40×. **B**) Neuraminidase (NA) reaction in total cellular protein extract by a dot blot assay: a) Mock infected cells; b) MuV-infected cells; c) pcDNA-3.1-transfected cells; d) pcDNAHN176-transfected Vero cells. **C) **% of hemoglobin (Hb) released after the HD. The Hb absorbance of MuV-infected cells was considered as 100% and was used to calculate the % of Hb for the different cells. **D**) Comparison of the NA activity in MuV-infected cells, pcDNA3.1-transfected cells and pcDNAHN176-transfected Vero cells by dot blot and spectrophotometric methods.

To analyze if the expressed HN176 polypeptide retained the main biological activities of the complete HN protein, the hemagglutinin property was evaluated by a hemadsorption (HD) reaction with sialic acid receptors present on red cell membranes [25]. MuV-infected cells showed an HD reaction characterized by the presence of clumps of red cells on them (Figure 3Ab), while no reactivity was found in pcDNA3.1-transfected cells (Figure 3Ac) or mock-infected cells (Figure 3Aa). In contrast, an HD reaction was also observed in the pcDNAHN176-transfected cells, although the intensity of the HD reaction was lower than in MuV-infected cells, but the erythrocytes were clearly observed (Figure 3Ad). To verify the specificity of the HD reaction, the amount of red cells adsorbed to the cells was measured using the quantitative colorimetric determination of hemoglobin concentration according to the method described by Drabkin, 1935 [26]. No hemoglobin was detected in pcDNA3.1-transfected cells or mock-infected cells, but the pcDNAHN176-transfected cells (Figure 3C) showed 40% of the hemoglobin concentration in comparison with the hemoglobin detected in MuV-infected cells. Furthermore, the HD reaction was not observed in MuV-infected Vero cells or in the pcDNAHN176-transfected cells when a hyperimmune anti-MuV serum was used, supporting the specificity of the HD reaction (data not shown).

To analyze if the HN176 polypeptide also had a neuraminidase activity (NA), total protein extracts obtained from pcDNAHN176-transfected cells and from MuV-infected cells were tested by a dot and spectrophotometric assays using 2'-(4 methyliferyl)-α D-N-acetylneuraminic acid (MU-NANA) as a substrate. Both cellular extracts displayed neuraminidase activity (Figures. 3B and 3D); however, the NA activity in the pcDNAHN176-transfected cells was lower, 38.93% and 24.4% by dot and spectrophotometric methods, respectively, compared to the NA activity in the MuV-infected cells.

All of these results indicated that the pcDNAHN176-transfected cells presented both activities, HD and NA, of the HN MuV complete protein.

### Immunogenic properties of the HN 176 polypeptide

#### Rabbit's sera

**Figure 4:**
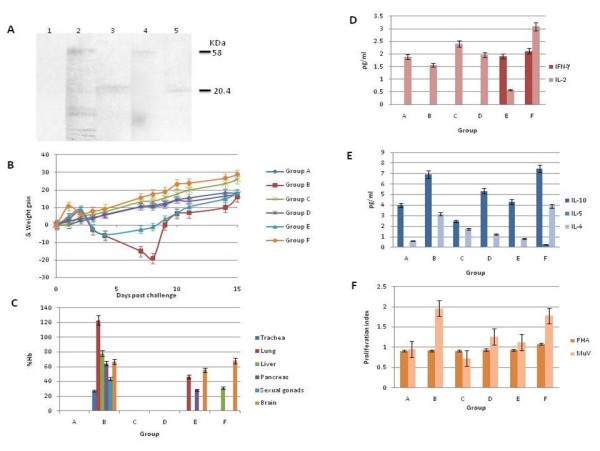
**Immunological properties of the pcDNAHN176-construct**. **A) **Detection of the HN176 polypeptide and HN protein by Western Blot. Lane 1) Negative control, uninfected cells and sera from pcDNAHN176-immunized rabbits; Lane 2) MuV-infected-Vero cells and anti-MuV serum; Lane 3) pcDNAHN176-transfected cells and anti-MuV serum; Lane 4) MuV-infected-Vero cells and sera from pcDNAHN176-immunized rabbits; Lane 5) pcDNAHN176-transfected cells and sera from pcDNAHN176-immunized rabbits. The upper arrow indicates in lane 2 and 4 the position of the complete viral HN protein, and the lower arrow indicates the position of the HN176 polypeptide in lanes 3 & 5. **B) **Body gain weight of hamsters immunized and challenged with MuV. Group A, animals without immunization and uninfected; Group B, viral control group (animals without immunization and challenged with MuV); Group C, animals immunized with pcDNA3.1 without challenge; Group D, animals immunized with pcDNAHN176-construct without challenge; Group E, animals immunized with pcDNA3.1 and challenged with MuV; Group F, animals immunized with pcDNAHN176 and challenged with MuV. **C) **Virus isolation from different organs of the hamsters groups. MuV was detected by HD, quantifying the amount of Hb. **D) **Detection of IL associated to Th1 response in the hamsters groups. **E) **Detection of IL associated to Th2 response in the hamsters groups. ILs were measured using the Luminex System (Invitrogen ^®^). **F) **Lymphoproliferation index of spleen cells obtained from the hamsters groups. Cell proliferation was measured by MTT method.

Sera obtained from rabbits immunized with the pcDNAHN176-construct had a 50% titer of neutralizing antibodies of 10^3.75 ^for both the Urabe and Jeryl Lynn MuV strains (Table 1). Furthermore, western blot analysis confirmed that the sera from rabbits immunized with the construct were able to recognize the complete 58 KDa viral HN protein, and also the 20.4 KDa polypeptide expressed by the pcDNAHN176-transfected cells (Figure 4A lanes 4 and 5). These assays showed that pcDNAHN176-immunization induced an immune response that recognized the complete HN protein and neutralized MuV infection.

**Table 1:** Titer of neutralizing antibodies against MuV *

	Hamsters sera						Rabbits sera	
Group	A Unimmunized/ Unchallenged	B Unimmunized/ Challenged (viral control group)	C 3.1 Immunized/ Unchallenged	D HN Immunized/ Unchallenged	E 3.1 Immunized/ Challenged	F HN Immunized/ Challenged	Urabe AM9	JL

NI_50_	ND	10^3.26^	ND	10^2.20^	10 ^2.97^	10 ^3.36^	10^3.75^	10^3.75^

*Expressed as the reciprocal of the highest serum dilution that protects 50% of the inoculated cell cultures.

#### Hamster protection

Figure 4 presents the body weight gain after MuV challenge in the six hamsters groups. It is clearly shown that the non-immunized (group B) or the pcDNA3.1-immunized (group E) animals experienced a mean weight loss of 5-20% of their original body weight 4-7 days after MuV challenge. On the contrary, the pcDNAHN176-immunized group (F) gained body weight compared with the non-challenged groups (A, C, D). To confirm MuV infection in the challenged groups, several organs were removed and used for MuV isolation in Vero cells, and the virus was detected by HD reaction. MuV was isolated from all three challenged groups (B, E, and F). Trachea, lung, liver, pancreas, sexual gonads and brain from the viral control group (B) and group G were positive for MuV isolation; in contrast, only the liver and brain from the pcDNAHN176-immunized group (F) were positive for viral isolation (Figure 5C).

In all of the animal groups challenged with MuV (B, E, F), neutralizing antibodies against MuV were detected, and in all of them, the titers were similar (Table 1). DNA immunization with the pcDNAHN176-construct (group D) induced neutralization antibodies, but the titer was low, and after viral challenge, the titer of neutralizing antibodies was similar to that of the viral control group (B).

The lymphoproliferation assay (Figure 4F) showed that the group immunized with the pcDNAHN176-construct (Group D) induced a higher and specific response against MuV compared to the pcDNA3.1-immunized group (C), whose response to MuV was lower than the response to the PHA mitogen. However, the lymphoproliferative response to MuV in group F (construct immunized/MuV challenged) was similar to the response found in group B (viral control group), indicating that MuV infection induced a certain level of specific lymphoproliferative response, which was not increased, even in animals that were immunized before viral infection.

Cytokines like IFNγ were detected only in the pcDNA3.1- or pcDNAHN176-immunized/challenged groups (E, F), and in both groups, the IFNγ concentration was similar (Figure 4D). IL-2, IL 10 and IL 4 were detected in all of the groups, but only IL 5 was detected in group F (Figure 4E). All of these results suggested that pcDNAHN176-immunized animals were capable of responding to MuV infection by inducing both the Th1 and Th2 immune specific responses and probably secretion of IgA in the mucosa.

## Discussion

MuV HN protein is a highly relevant protein in the viral infectious cycle. It is responsible for viral interaction with cellular receptors and, in fact, is the main viral antigenic determinant [23]. In this paper, a highly conserved and immunogenic region of the MuV HN gene was chosen for cloning based on our bioinformatics analysis carried out with several MuV genotypes. The region was located from 817 to 1383 nucleotides in the Urabe MuV strain, which potentially encodes a 176 aa polypeptide (HN176) corresponding to the amino acid positions 255 to 431 of the HN protein.

After cloning the HN region in the eukaryotic expression vector pcDNA3.1, which is commonly used for high-level stable expression in mammalian cells [27-30], its expression was analyzed in Vero-transfected cells. We found that the pcDNAHN176-construct over expressed the corresponding RNA and polypeptide. The HN176 polypeptide was demonstrated by immunochemistry and immunofluorescence methods using a reference hyperimmune serum anti-MuV, indicating that at least one of the antigenic HN epitopes previously reported at positions 265-288, 213-372 and 352-360 was exposed [18,31,32].

Knowing that the HN176 polypeptide is including the region that has been suggested to be involved in the NA activity [33], this activity was measured in total protein extracts. We verified by two methods that the NA activity was present in the pcDNAHN176-transfected cells. However, this NA activity was lower compared to the activity in MuV-infected cells. Because the main receptor binding domain of the MuV HN protein has been proposed to be located at the same site as the neuraminidase activity [34], the HN176 polypeptide's ability to recognize sialic receptors on red cells was evaluated in the construct-transfected cells using HA and HD reactions. No HA was detected, but an HD reaction was positive in the pcDNAHN176-transfected cells. This result was unexpected because the construct did not have a signal peptide to sort the HN176 polypeptide into the cellular membrane. However, the specificity of HD reaction was verified by blocking the reaction with anti-MuV serum and, furthermore, by detecting the amount of hemoglobin released from the cells using Drabkin 's method. Nevertheless, the NA and HD activities in the pcDNAHN176-transfected cells were lower than in MuV-infected cells, and none of these activities were detected in the uninfected control or pcDNA3.1-transfected cells. The low NA and HD activities were probably due to an incomplete NA site in the HN176 polypeptide. However, these results support the proposal that the pcDNAHN176-transfected cells were capable of expressing the HN176 polypeptide in a correct folding structure and exporting it to the surface such that it was accessible to the erythrocytes or the antibodies to inhibit the HD reaction. We do not know the mechanism by which the peptide could be expressed on the cell surface, but other groups using the same pcDNA3.1 vector have reported that some proteins lacking the signal peptide are sorted into the cellular membrane [35,36]. Some of those proteins have shown the RXLXEQ motif, which has been associated with ER exportation [37]. By bioinformatic analysis of our HN176 polypeptide, it seemed to contain the RXLXEQ motif in positions 162-167 (data not shown).

Knowing that DNA vaccines can induce both humoral and cell-mediated immune responses against many different antigens and that the immune response may depend more on their ability to produce the mature protein in an appropriate conformation than on whether the protein is membrane-anchored or soluble and whether it is targeted for secretion by conventional mechanisms [38], we initially evaluated the immunogenic properties of the pcDNAHN176-construct by immunization of rabbits using intradermal inoculation into the ear pinnae. Usually, DNA vaccines are administered through an intramuscular route; however, intradermal inoculation has been a very successful route for DNA plasmids [39]. Murine ear pinnae immunization has shown to be an excellent site for initiating immune responses with DNA vaccines [40]. Mechanism(s) accounting for the superiority of the ear pinnae as a vaccination site are ascribed to its unique immunological features, which focus the concentration of processed antigen in a restricted area that is connected with a major draining lymph node. It is thought that the concentration of processed antigen results in an enhanced stimulation of T lymphocytes by antigen-loaded dendritic cells [40]. We found that the rabbit's sera neutralized the Urabe and the Jeryl Lynn MuV strains. Furthermore, the sera neutralized both MuV strains to the same extent, even though these strains belonged to a different MuV genotype. The first one was the B genotype, and the last one was the A genotype, indicating that immunization with the pcDNAHN176-construct induced specific antibodies capable of neutralizing two different MuV genotypes. Additionally, the antibodies induced by the construct immunization specifically reacted with the complete MuV HN protein and the HN176 polypeptide, as observed by western blot assay (Figure 4A).

To confirm the immunogenic properties of the construct, a MuV hamster intranasal infection was used. Animals were split in six groups and intradermally immunized in the ear pinnae with either the DNA-construct or pcDNA3.1 vector. First, the body weight of the animals was measured daily, and we found that the MuV control (Group B), pcDNA3.1-immunized and MuV challenged groups (Group E) presented a weight loss, while the animals immunized with the pcDNAHN176-construct and challenged with MuV (Group F) showed a similar gain in weight to the non-viral infected groups (Groups A, C, D) (Figure 4B). To verify the viral infection, samples of different organs were analyzed for MuV by isolation in Vero cells, and we found that all of the samples from the viral control group (Group B) were positive, but only two positive samples were detected in the construct-immunized group (Group F). Therefore, these results showed that immunization with the DNA construct containing the 567 nt region of the HN gene ameliorated MuV infection, probably by reducing viral dissemination to different organs.

To evaluate the effect of the pcDNAHN176-construct vaccination on cellular responses, we measured lymphocyte proliferation in response to specific MuV antigens (Figure 4F). In the construct-immunized group (Group F), there was a higher proliferation index when the cells were stimulated with MuV than when using a mitogen (PHA). However, the lymphoproliferation index was very similar to the viral control group (B).

Spleen cells from group F showed that MuV-stimulated cultures contained high levels of IL-2 and γ-interferon with little IL-4, indicating that intradermal DNA vaccination in the ear pinnae area elicited Th1-like cytokine responses. However, IL 5 was detected, suggesting a mixed-phenotype or Th2-like response. Lower levels of IFNγ and IL2 were detected in the pcDNA3.1-immunized and challenged hamsters (Group E). This unspecific induction by pcDNA3.1 immunization in hamsters has been previously reported [35]. Plasmid DNA vaccines, when injected intramuscularly or intradermally, induce a Th1 response due to the vector CpG motifs that stimulate the production of IL-12, which favors the activation of Th1 lymphocytes [41]. DNA vaccines have been shown to induce antigen-specific IFN-γ-secreting Th1 cells, which are detectable in the spleen or lymph nodes [42], and also generate Th2 or mixed Th1/Th2 type responses [42,43]. In this report, we found that the pcDNAHN176-construct was capable of inducing both the Th1 and Th2 responses, and the ear pinnae immunization seemed to produced better results than intramuscular (IMI) immunization because the IMI did not reduce the weight loss and MuV was detected in all of the analyzed organs (data not shown).

Therefore, the pcDNAHN176-construct could be a good candidate for use as a DNA vaccine. This proposal is also supported by the bioinformatic analysis we carried out with 81 strains of nine known different MuV genotypes and 13 strains with unknown genotypes deposited in GenBank [44], which confirmed that the HN176 region is highly conserved among the different MuV types.

## Conclusions

The pcDNAHN176-construct expresses a polypeptide in Vero cells that conserves the main biological properties of the HN protein. The construct immunization in rabbits and hamsters was capable of inducing a specific immune response against MuV. The results are very encouraging for a MuV DNA vaccine, which could be very useful against the different MuV genotypes. Therefore, it is important to carry out more studies to evaluate the pcDNAHN176-construct against more MuV genotypes, determine how long the immune response lasts and improve its immunogenic properties to obtain a long-lasting immune response before it can be proposed as a new MuV vaccine.

## Methods

### Selection and cloning of a region from the HN gene

The nucleotide sequences of the mump virus HN gene were obtained from GenBank [44] (accession numbers: X93178, X93179, X93180, X93181, X15284, X98875, X98874, X93177, D86170) and translated *in silico *using the ExPASy Proteomics Server [45]. Nucleotide and amino acid sequences were aligned using ClustalW to search for highly conserved regions among the different MuV strains. The protein antigenic properties of the protein were evaluated using the ANTHEPROT software [46] and the antigenicity scale described by Parker et al. [47]. We found 27 highly conserved regions and 30 antigenic regions, six of them represented the most antigenic segments.

Based on those analyses, a set of oligonucleotide primers (HN-sense 5' CGCGGATCCAGCTGCTCAATTGCAACAGTCCCT 3' and HN-antisense 5' GGGGTACCGAGTTCATACGGCCACCAGCT 3') was designed to amplify the region from nucleotides 817 to 1383 of the HN gene.

### Virus, cells and vectors

The Urabe Am-9 mumps virus strain was grown in chicken embryo fibroblast cell cultures using M-199 supplemented with 10% newborn calf serum and purified by the polyethylene glycol precipitation method.

Viral RNA was extracted with TRIzol (GIBCO BRL^®^) according to manufacturer's protocol, and reverse transcribed into cDNA using 3 μg of RNA, 1 μl of the HN sense primer (200 μM), 6 μl of RT buffer (10×), 4 μl of DDT (0.1 M), 4 μl of dNTPs (10 mM), 1 μl of RNasin and 1 μl of RT (200 U/ml, Super Script). The reaction was held at 42°C for 50 min.

Five μl of the RT product was PCR amplified using the Taq PCR Core Kit (QIAGEN^®^) and the HN-sense and HN-antisense primers at 92°C for 5 min, followed by 30 cycles of 45 s at 94°C, 45 s at 66°C and 45 s at 72°C, with a final extension at 72°C for 7 min.

The PCR product was purified and directly cloned in frame into the *Kpn*I and *Bam*HI sites of the pcDNA3.1(+) expression vector (Invitrogen^®^). The integrity and orientation of the construct (pcDNA-HN176) were verified by restriction analysis and automatically sequenced using the dideoxynucleotide chain-termination method [48].

### Expression of the HN insert

Vero cells grown in M199 supplemented with 10% of newborn calf serum were transfected with the pcDNAHN176-construct or pcDNA3.1 using the PolyFect Transfection Reagent (QIAGEN^®^) following the manufacturer's instructions. Transfected cells were selected with 0.8 mg/ml of G418 (Invitrogen). Cellular RNA from transfected and non-transfected cells was obtained using the TRIzol (GIBCO BRL^®^) method. Cellular RNA (25 ng) was reverse transcribed using the Sensiscript RT Kit (QIAGEN^®^), and the HN-antisense primer and the RT product was PCR amplified using the Taq PCR Core Kit (QIAGEN^®^) and both primers, HN-sense and HN-antisense. The PCR conditions were the same as described above. The PCR product was analyzed by 1% agarose gel electrophoresis followed by staining with ethidium bromide.

#### Immunochemistry

Transfected Vero cells were grown on cover slips and fixed with 4% paraformaldehyde at room temperature. Cells were washed with PBS for 5 min and blocked with albumin 1%-tween 20 (0.001%) overnight at 4°C. Then the cells were washed again and incubated with a reference anti-mumps antibody (horse hyperimmune serum, kindly donated by the Centers for Disease Control and Prevention, (CDC, USA) at 4°C overnight. Following, the cells were incubated with a biotinylated anti-horse phosphatase alkaline antibody (Jackson Immuno Research) for 2 h at room temperature. Finally, cells were incubated with APPurple (Intergen^®^) for 15 min at room temperature.

#### Immunofluorescence

Transfected cells were grown and fixed as describe above, washed with PBS for 5 min and blocked with 1% albumin overnight at 4°C. Then the cells were overnight incubated with rabbit anti-MuV serum at 4°C, and afterwards, incubated with an anti-rabbit fluorescent antibody (Jackson Immuno Research^®^) for 2 h at room temperature. Finally, the cells were observed under a fluorescent microscope.

In both immunoassays, Vero cells infected with MuV at 0.2 MOI and incubated for 72 h were used as an HN positive control.

### Biological activities of the HN 176 polypeptide

#### Hemadsorption (HD) assay

The transfected Vero cells were grown in microplates. Two days later, the cellular medium was removed, the cells were washed with PBS and a suspension of 4% guinea pig red cells was added to the cells for 1 h at 4°C. The cells were extensively washed with PBS and observed under an inverted microscope [25]. The amount of bound red cells was calculated by measurement the hemoglobin on the cells using the hemoglobincyanide method described by Drabkin, 1935 [26]. The amount of hemoglobin found in the MuV-infected cells was considered as 100%, and as proportional to the amount of erythrocytes absorbed to the cells. The specificity of the HD assay was verified by HD inhibition using the reference horse hyperimmune anti-MuV serum. As a positive control for the HD and HDI assays, Vero cells infected with MuV at 0.2 MOI and incubated for 72 h were used.

#### Neuraminidase (NA) assay

A total cellular protein extract was obtained from the transfected Vero cells, which was concentrated by centrifugation and resuspended in PBS-triton 100 (0.01%) in the presence of a protease inhibitor cocktail (SIGMA). Proteins were precipitated overnight using cold acetone at -20°C and pelleted at 12,000 × *g*. The protein concentration was determined using Bradford's method [49], and neuraminidase activity was measured by the dot assay using the synthetic substrate 2'-(4-methylumbelliferyl)-α D-N-acetylneuraminic acid (MU-NANA) according to the method described by Moncla and Braham, [50]. Additionally, a spectrophotometric assay was used. In both assays, the relative neuraminidase activity was expressed as a percentage of the NA activity observed in similar extracts obtained from MuV-infected cells.

### Immunogenic properties

All animal experiments were carried under the supervision of the Institutional Bioethical Committee and the Head of the animal house facilities.

#### Rabbit Immunization

Two eight-week old rabbits were immunized into the pinnea area of the ear via intradermal injection using 100 μg of pcDNAHN176-construct DNA. Another two rabbits were immunized with the pcDNA3.1 plasmid. Eight days later, a second boost immunization was applied in the same zone. As a positive control, a rabbit was immunized with MuV in complete Freud adjuvant, and a week later, a 2^nd ^boost with incomplete Freud adjuvant was applied. At day twenty-three, the animals were euthanized, and serum was collected and concentrated by precipitation with ammonium sulfate [51]. Sera were used for viral neutralization assays and western blot analyses.

#### Neutralization assay

Two MuV strains, the Urabe and the Jeryl Lynn strains, were used in the assay. The NT antibody titer was calculated using Kärber's formula and expressed as the 50% neutralizing endpoint dilution of the serum.

#### Western Blot

To verify that the antibodies specifically recognized the viral HN protein, cellular extract obtained from MuV-Vero infected cells or pcDNAHN176 transfected cells were separated by 16% SDS-PAGE. The proteins were electrophoretically transferred onto nitrocellulose membranes at 100 mA for 4 h. After washing the membranes with distilled water, they were blocked with PBS-Albumin (1%) overnight at 4°C. Rabbit serum diluted 1:100 was added, followed by incubation overnight at 4°C. The bound antibodies were detected by horseradish peroxidase-conjugated anti-rabbit IgG (Zimed ^®^).

#### Hamster immunization and viral challenge

Five-week old hamsters used in the assays were divided in six groups: Group A, animals without immunization and without challenge; Group B, animals without immunization and challenged with MuV (viral control group); Group C, animals immunized with pcDNA3.1 without challenge; Group D, animals immunized with pcDNAHN176 without challenge; Group E, animals immunized with pcDNA3.1 and challenged with MuV; and Group F, animals immunized with pcDNAHN176-construct and challenged with MuV.

Hamsters were immunized intradermically into the ear pinnae using 100 μg of DNA from the pcDNAHN176-construct or pcDNA3.1 plasmid. Seven days later, a second boost immunization was applied in the same zone. A week after the last immunization, the hamsters were intranasally infected with 100 μl of 10^6.8 ^TCID_50_/ml Urabe AM9 MuV. The animals were euthanized fourteen days after the viral challenge to obtain serum, liver, pancreas, sexual gonads, lungs, trachea and brain. The organs were macerated and used to infect Vero cells. Five days after infection, an HD assay was realized.

The spleen cells were used for a lymphoproliferation assays. Briefly, 5 × 10^5 ^spleen cells were cultivated in 96-well dishes and stimulated with 5 × 10^5 ^TCID50/ml of MuV or 3 μg of PHA. Seventy-two hours after stimulation, the cell proliferation was measured using the MTT method.

Another sample of spleen cells was stimulated with MuV as described above and incubated for 72 hours. The supernatant was used to measure several cytokines using the Mouse Th1/Th2 Six-Plex Antibody Bead Kit Luminex System (Invitrogen ^®^) according to the manufacturer's instructions. Sera of the animals were used to measure the neutralizing antibodies.

All of the animals were observed and weighed every day.

## Competing interests

The authors declare that they have no competing interests.

## Authors' contributions

EH: Obtained the pcDNAHN176-construct. Biological and immunological evaluation of the pcDNAHN176-construct in the hamster model. Manuscript writing. PB: Immunological evaluation of the pcDNAHN176-construct in rabbits. RH: Bioinformatic analysis of the HN176 peptide. AM: Bioinformatic analysis of the HN MuV protein to detected highly conserved and immunogenic regions. GP-I: Design of the pcDNAHN176-construct. BB: Conception and design of the assays, and final manuscript revision.

All authors have read and approved the final manuscript
